# Cadherin-16 (CDH16) immunohistochemistry: a useful diagnostic tool for renal cell carcinoma and papillary carcinomas of the thyroid

**DOI:** 10.1038/s41598-023-39945-2

**Published:** 2023-08-09

**Authors:** Maximilian Lennartz, Henrietta Csomós, Viktoria Chirico, Sören Weidemann, Natalia Gorbokon, Anne Menz, Franziska Büscheck, Claudia Hube-Magg, Doris Höflmayer, Christian Bernreuther, Niclas C. Blessin, Patrick Lebok, Guido Sauter, Stefan Steurer, Eike Burandt, David Dum, Till Krech, Ronald Simon, Sarah Minner, Frank Jacobsen, Till S. Clauditz, Andreas M. Luebke, Abdul Khalid Siraj, Fouad Al-Dayel, Khawla S. Al-Kuraya, Andrea Hinsch

**Affiliations:** 1 Institute of Pathology, University Medical Center Hamburg-Eppendorf, Martinistr. 52, 20246 Hamburg, Germany; 2 Institute of Pathology, Clinical Center Osnabrueck, Osnabrueck, Germany; 3 Department of Human Cancer Genomic Research, Research Center, King Faisal Specialist Hospital and Research Center, Riyadh, Saudi Arabia

**Keywords:** Cancer, Tumour biomarkers

## Abstract

Cadherin-16 (CDH16) plays a role in the embryonal development in kidney and thyroid. Downregulation of CDH16 RNA was found in papillary carcinomas of the thyroid. To determine the expression of CDH16 in tumors and to assess the diagnostic utility a tissue microarray containing 15,584 samples from 152 different tumor types as well as 608 samples of 76 different normal tissue types was analyzed. A membranous CDH16 immunostaining was predominantly seen in thyroid, kidney, cauda epididymis, and mesonephric remnants. In the thyroid, CDH16 staining was seen in 100% of normal samples, 86% of follicular adenomas, 60% of follicular carcinomas, but only 7% of papillary carcinomas (p < 0.0001). CDH16 positivity was frequent in nephrogenic adenomas (100%), oncocytomas (98%), chromophobe (97%), clear cell (85%), and papillary (76%) renal cell carcinomas (RCCs), various subtypes of carcinoma of the ovary (16–56%), various subtyped of carcinomas of the uterus (18–40%), as well as in various subtypes of neuroendocrine neoplasms (4–26%). Nineteen further tumor entities showed a weak to moderate CDH16 staining in up to 8% of cases. Our data suggest CDH16 as a potential diagnostic marker—as a part of a panel—for the identification of papillary carcinomas of the thyroid, nephrogenic adenomas, and the distinction of renal cell tumors from other neoplasms.

## Introduction

Cadherin-16 (CDH16) is a calcium-dependent, membrane bound cell-adhesion protein with a role in the formation of tubular epithelial structures in only a few organs. In the kidney, CDH16 promotes the formation of renal tubuli^1^ and shows persistent high-level expression in the adult kidney. Accordingly, CDH16 has also been named kidney specific cadherin (ksp-cadherin)^1,2^. However, CDH16 also plays a role in the development of thyroid follicles, and it is expressed in all follicular cells of the adult thyroid gland^3^.

RNA expression data suggest that CDH16 expression in normal tissues may be limited to the kidney, the thyroid and only few other tissues^1,4–9^. In the kidney and the thyroid, reduced expression of CDH16 has been linked to the development of cancer^4,9^. Only a small number of studies have used immunohistochemistry to analyze CDH16 expression in cancer and these were limited to renal cell carcinomas (RCC). CDH16 protein expression has been described to occur in 0–30% of clear cell RCC^10–15^, 0–29% of papillary RCC^10–13,16^, 5–100% of chromophobe RCC^10–15,17^, and in 0–95% of oncocytomas of the kidney^10–14,17^. Data from publicly available RNA databases suggest that CDH16 expression can—less commonly—also be found in other tumor entities including cervical, endometrial, and ovarian cancers^4,9,18–20^.

Given the predilection of CDH16 RNA expression to the kidney and the thyroid, CDH16 antibodies may be useful for the distinction of renal or thyroidal neoplasms from other cancers. However, immunohistochemical analyses of CDH16 expression are so far lacking for most tumor entities. To assess the diagnostic utility of immunohistochemical CDH16 expression analysis, the protein was evaluated in more than 15,800 tumor tissue samples from 152 different tumor types and subtypes as well as in 76 non-neoplastic tissue categories by immunohistochemistry (IHC) in a tissue microarray format in this study.

## Results

### Technical issues

A total of 13,424 (88.1%) of 15,584 tumor samples and more than 540 normal samples were interpretable in our TMA and large section analysis. Non-interpretable samples demonstrated lack of unequivocal tumor cells or absence of tissue in the respective TMA spots.

### CDH16 in normal tissues

**Figure 1:**
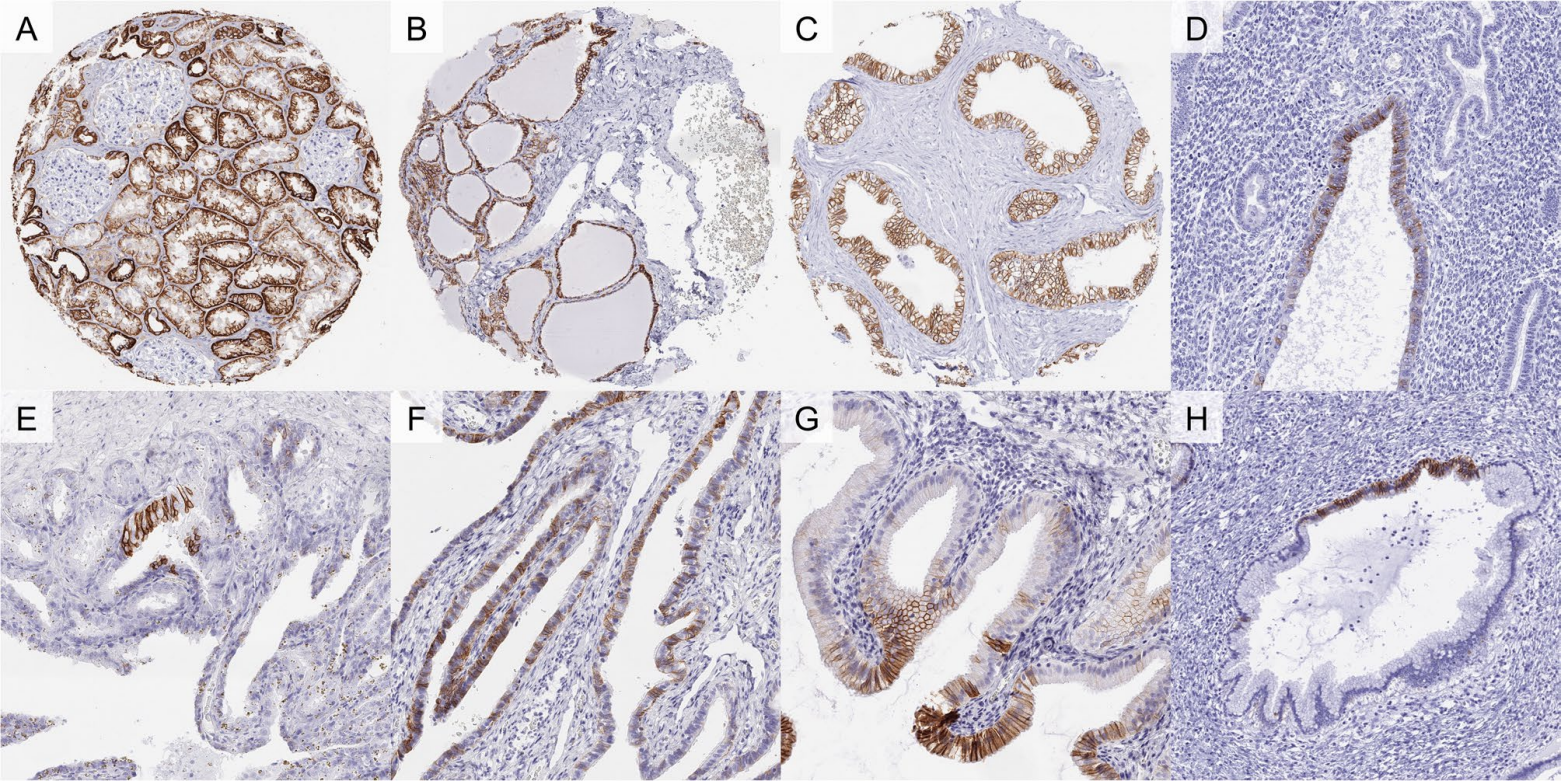
CDH16 immunostaining of normal tissues. In the kidney, CDH16 staining is predominantly basolateral and stronger in distal tubuli and collecting ducts than in proximal tubuli (**A**). In follicular cells of the thyroid (**B**) and epithelial cells of the cauda epididymis (**C**), a diffuse strong membranous staining is seen. A membranous staining of individual cells, groups of cells, or of individual glands can be seen in the endometrium (**D**), seminal vesicles (**E**), the fallopian tube (**F**), gallbladder epithelium (**G**), and the endocervix (**H**).

CDH16 immunostaining was predominantly seen in the kidney, thyroid and the epididymis. In the kidney, CDH16 immunostaining was stronger in proximal tubuli and in collecting ducts than in distal tubuli. The staining pattern was membranous (predominantly basolateral) and also cytoplasmic. In the thyroid, a strong membranous CDH16 staining occurred in follicular cells. In the epididymis, a predominantly membranous but also cytoplasmic staining was preferably seen in epithelial cells of the cauda while staining was absent or markedly weaker in the caput. A small fraction of epithelial cells, often arranged in nests or groups, showed a moderate to strong CDH16 staining in seminal vesicles. In some (but not all) analyzed samples, a focal weak to moderate membranous and cytoplasmic staining of individual cells, groups of cells or individual glands was seen in gallbladder epithelium, endometrium, and in the fallopian tube. Large section analyses also identified a strong CDH16 staining in Wolffian (mesonephric) duct remnants of the fallopian tube and in scattered cells, small groups of cells or of a limited number of glands in endocervical epithelium. Representative images of normal tissues are shown in Fig. 1. All these findings were obtained by using the monoclonal rabbit recombinant antibody MSVA-516R and the monoclonal rabbit antibody EPR13090, although EPR13090 resulted in a markedly less favorable signal to noise ratio. A cytoplasmic staining of gastric glands and of adrenocortical cells was only seen by EPR13090, but not by MSVA-516R, and was thus considered an antibody specific cross-reactivity. A comparison of antibody staining is shown in Supplementary Fig. 1.

### CDH16 in cancer

**Figure 2:**
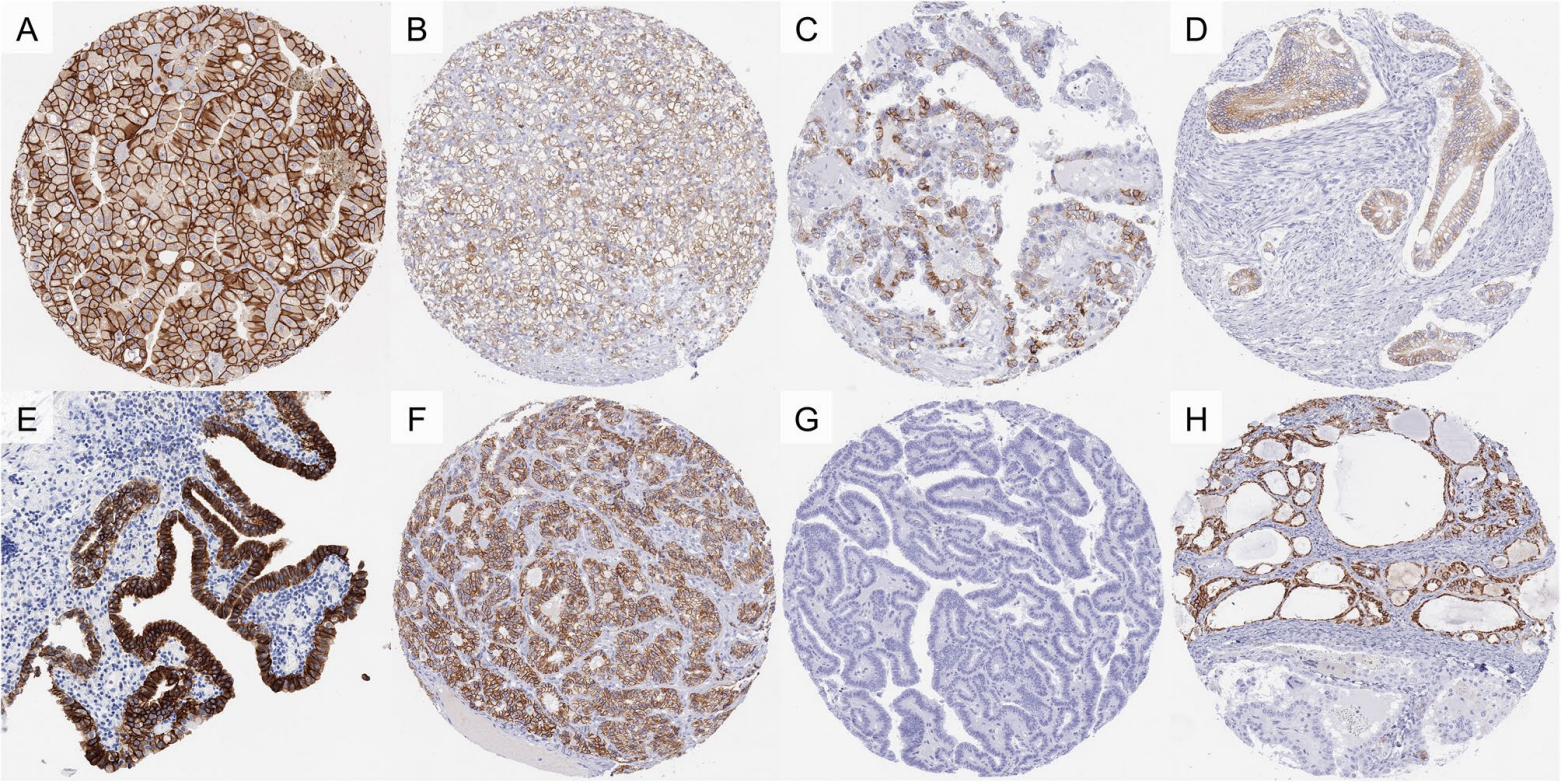
CDH16 immunostaining in cancer. The panels show a predominantly membranous CDH16 immunostaining of variable intensity in samples from a chromophobe (**A**) and a clear cell renal cell carcinoma (**B**), a clear cell carcinoma of the ovary (**C**), an adenocarcinoma of the cervix uteri (**D**), a nephrogenic adenoma (**E**), and a follicular adenoma of the thyroid (**F**). Samples from CDH16 negative papillary carcinomas of the thyroid are depicted in (**G**) and—adjacent to CDH16 positive normal thyroid follicles—in (**H**).

**Figure 3:**
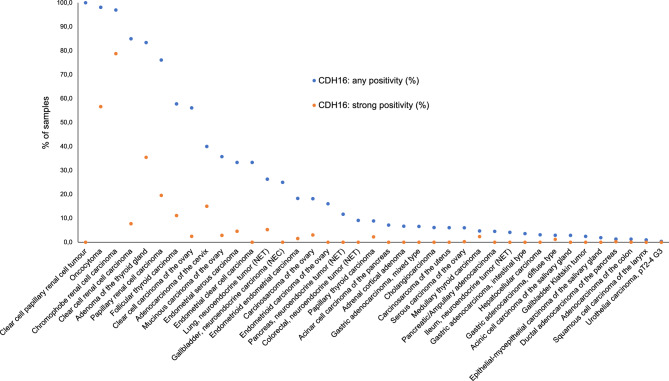
Ranking order of CDH16 immunostaining in tumors. Both the frequency of positive cases (blue dots) and the frequency of strongly positive cases (orange dots) are shown.

CDH16 immunostaining was detectable in 1074 (8.0%) of the 13,424 analyzable tumors, including 476 (3.5%) with weak, 309 (2.3%) with moderate, and 289 (2.2%) with strong immunostaining. Overall, 40 (26.3%) of 152 tumor categories showed detectable CDH16 expression and 19 (12.5%) tumor categories included at least one case with strong positivity (Table 1). Representative images of CDH16 positive tumors are shown in Fig. 2. The highest rate of positive staining was found in renal cell carcinomas and follicular neoplasms of the thyroid, followed by several tumor entities of the female genital tract and of neuroendocrine neoplasms. CDH16 positivity was particularly frequent in nephrogenic adenomas (100%), oncocytomas (98%), chromophobe (97%), clear cell (85%), and papillary (76%) renal cell carcinomas (RCCs), follicular adenomas (86%) and follicular carcinomas (60%) of the thyroid, clear cell (56%), mucinous (36%), and endometroid (16%) carcinoma and carcinosarcoma (18%), of the ovary, adenocarcinoma of the cervix uteri (40%), serous (33%), clear cell (33%), and endometroid carcinoma (18%) of the endometrium as well as in various subtypes of neuroendocrine neoplasms (4–26%). In thyroid tissues, there was a significant decrease of CDH16 positivity from normal thyroid (8 of 8 positive, 100%) to follicular adenomas (81 of 94, 86.2%), follicular carcinomas (40 of 67, 59.7%) and papillary carcinomas (14 of 212, 6.6%; (p < 0.0001). In renal cell tumors, CDH16 positivity was significantly more frequent in oncocytoma (104 of 106 positive, 98%) and chromophobe cancers (64 of 66, 97%) than in clear cell (384 of 452, 85%) and papillary renal cell carcinomas (105 of 138, 76%; p < 0.0001). A CDH16 positivity was also seen in up to 8% of cases in 19 additional tumor categories but the staining was only weak to moderate in the vast majority of these cases. An additional large section analysis revealed a CDH16 positivity in all 12 nephrogenic adenomas of the urinary bladder (9 strong, 3 moderate) and absence of CDH16 staining in 3 peritoneal and 2 mesotheliomas of the tunica albuginea. A ranking order of tumor categories according to their rate of CDH16 positive and strongly positive cases is given in Fig. 3.

**Table 1:** CDH16 immunostaining in human tumors.

	Tumor entity	On TMA (n)	CDH16 IHC result				
			Analyzable (n)	Negative (%)	Weak (%)	Moderate (%)	Strong (%)
Tumors of the skin	Pilomatrixoma	35	31	100.0	0.0	0.0	0.0
	Basal cell carcinoma	88	74	100.0	0.0	0.0	0.0
	Benign nevus	29	27	100.0	0.0	0.0	0.0
	Squamous cell carcinoma of the skin	90	88	100.0	0.0	0.0	0.0
	Malignant melanoma	46	43	100.0	0.0	0.0	0.0
	Malignant melanoma Lymph node metastasis	86	76	100.0	0.0	0.0	0.0
	Merkel cell carcinoma	46	41	100.0	0.0	0.0	0.0
Tumors of the head and neck	Squamous cell carcinoma of the larynx	109	104	99.0	1.0	0.0	0.0
	Squamous cell carcinoma of the pharynx	60	58	100.0	0.0	0.0	0.0
	Oral squamous cell carcinoma (floor of the mouth)	130	126	100.0	0.0	0.0	0.0
	Pleomorphic adenoma of the parotid gland	50	48	100.0	0.0	0.0	0.0
	Warthin tumor of the parotid gland	104	103	100.0	0.0	0.0	0.0
	Adenocarcinoma, NOS (Papillary Cystadenocarcinoma)	14	12	100.0	0.0	0.0	0.0
	Salivary duct carcinoma	15	15	100.0	0.0	0.0	0.0
	Acinic cell carcinoma of the salivary gland	181	141	97.2	2.8	0.0	0.0
	Adenocarcinoma NOS of the salivary gland	109	63	100.0	0.0	0.0	0.0
	Adenoid cystic carcinoma of the salivary gland	180	124	100.0	0.0	0.0	0.0
	Basal cell adenocarcinoma of the salivary gland	25	25	100.0	0.0	0.0	0.0
	Basal cell adenoma of the salivary gland	101	99	100.0	0.0	0.0	0.0
	Epithelial-myoepithelial carcinoma of the salivary gland	53	53	98.1	1.9	0.0	0.0
	Mucoepidermoid carcinoma of the salivary gland	343	328	100.0	0.0	0.0	0.0
	Myoepithelial carcinoma of the salivary gland	21	21	100.0	0.0	0.0	0.0
	Myoepithelioma of the salivary gland	11	11	100.0	0.0	0.0	0.0
	Oncocytic carcinoma of the salivary gland	12	12	100.0	0.0	0.0	0.0
	Polymorphous adenocarcinoma, low grade, of the salivary gland	41	40	100.0	0.0	0.0	0.0
	Pleomorphic adenoma of the salivary gland	53	40	100.0	0.0	0.0	0.0
Tumors of the lung, pleura and thymus	Adenocarcinoma of the lung	196	185	100.0	0.0	0.0	0.0
	Squamous cell carcinoma of the lung	80	73	100.0	0.0	0.0	0.0
	Small cell carcinoma of the lung	16	14	100.0	0.0	0.0	0.0
	Mesothelioma of the pleura, epitheloid	39	34	100.0	0.0	0.0	0.0
	Mesothelioma of the pleura, other types	76	61	100.0	0.0	0.0	0.0
	Mesothelioma of the peritoneum	3	3	100.0	0.0	0.0	0.0
	Mesothelioma of the tunica albuginea	2	2	100.0	0.0	0.0	0.0
	Thymoma	29	29	100.0	0.0	0.0	0.0
Tumors of the female genital tract	Squamous cell carcinoma of the vagina	78	69	100.0	0.0	0.0	0.0
	Squamous cell carcinoma of the vulva	130	118	100.0	0.0	0.0	0.0
	Squamous cell carcinoma of the cervix	129	124	98.4	0.8	0.0	0.8
	Adenocarcinoma of the cervix	21	20	60.0	15.0	10.0	15.0
	Endometrioid endometrial carcinoma	236	197	81.7	15.7	1.0	1.5
	Endometrial serous carcinoma	82	66	66.7	21.2	7.6	4.5
	Carcinosarcoma of the uterus	48	33	93.9	6.1	0.0	0.0
	Endometrial carcinoma, high grade, G3	13	11	100.0	0.0	0.0	0.0
	Endometrial clear cell carcinoma	8	6	66.7	16.7	16.7	0.0
	Endometrioid carcinoma of the ovary	110	81	84.0	14.8	1.2	0.0
	Serous carcinoma of the ovary	559	502	94.0	5.4	0.4	0.2
	Mucinous carcinoma of the ovary	96	70	64.3	27.1	5.7	2.9
	Clear cell carcinoma of the ovary	50	41	43.9	41.5	12.2	2.4
	Carcinosarcoma of the ovary	47	33	81.8	12.1	3.0	3.0
	Granulosa cell tumor of the ovary	37	36	100.0	0.0	0.0	0.0
	Leydig cell tumor of the ovary	4	4	100.0	0.0	0.0	0.0
	Sertoli cell tumor of the ovary	1	1	100.0	0.0	0.0	0.0
	Sertoli Leydig cell tumor of the ovary	3	3	100.0	0.0	0.0	0.0
	Steroid cell tumor of the ovary	3	3	100.0	0.0	0.0	0.0
	Brenner tumor	41	39	100.0	0.0	0.0	0.0
Tumors of the breast	Invasive breast carcinoma of no special type	499	413	100.0	0.0	0.0	0.0
	Lobular carcinoma of the breast	192	150	100.0	0.0	0.0	0.0
	Medullary carcinoma of the breast	23	22	100.0	0.0	0.0	0.0
	Tubular carcinoma of the breast	20	19	100.0	0.0	0.0	0.0
	Mucinous carcinoma of the breast	29	28	100.0	0.0	0.0	0.0
	Phyllodes tumor of the breast	50	48	100.0	0.0	0.0	0.0
Tumors of the digestive system	Adenomatous polyp, low-grade dysplasia	50	49	100.0	0.0	0.0	0.0
	Adenomatous polyp, high-grade dysplasia	50	48	100.0	0.0	0.0	0.0
	Adenocarcinoma of the colon	2482	2169	98.7	0.9	0.3	0.0
	Gastric adenocarcinoma, diffuse type	176	171	97.1	1.2	0.6	1.2
	Gastric adenocarcinoma, intestinal type	174	166	96.4	3.0	0.6	0.0
	Gastric adenocarcinoma, mixed type	62	61	93.4	6.6	0.0	0.0
	Adenocarcinoma of the esophagus	83	80	100.0	0.0	0.0	0.0
	Squamous cell carcinoma of the esophagus	76	68	100.0	0.0	0.0	0.0
	Squamous cell carcinoma of the anal canal	89	84	100.0	0.0	0.0	0.0
	Cholangiocarcinoma	50	49	93.9	4.1	2.0	0.0
	Gallbladder adenocarcinoma	31	15	100.0	0.0	0.0	0.0
	Gallbladder Klatskin tumor	41	41	97.6	2.4	0.0	0.0
	Hepatocellular carcinoma	300	292	96.9	3.1	0.0	0.0
	Ductal adenocarcinoma of the pancreas	612	462	98.7	0.9	0.2	0.2
	Pancreatic/Ampullary adenocarcinoma	89	67	95.5	4.5	0.0	0.0
	Acinar cell carcinoma of the pancreas	16	14	92.9	7.1	0.0	0.0
	Gastrointestinal stromal tumor (GIST)	50	50	100.0	0.0	0.0	0.0
Tumors of the urinary system	Nephrogenic adenomas of the urinary bladder	12	12	0.0	0.0	25.0	75.0
	Non-invasive papillary urothelial carcinoma, pTa G2 low grade	177	142	100.0	0.0	0.0	0.0
	Non-invasive papillary urothelial carcinoma, pTa G2 high grade	141	119	100.0	0.0	0.0	0.0
	Non-invasive papillary urothelial carcinoma, pTa G3	219	180	100.0	0.0	0.0	0.0
	Urothelial carcinoma, pT2-4 G3	735	630	99.7	0.3	0.0	0.0
	Squamous cell carcinoma of the bladder	22	21	100.0	0.0	0.0	0.0
	Small cell neuroendocrine carcinoma of the bladder	22	22	100.0	0.0	0.0	0.0
	Sarcomatoid urothelial carcinoma	25	24	100.0	0.0	0.0	0.0
	Urothelial carcinoma of the kidney pelvis	62	57	100.0	0.0	0.0	0.0
	Clear cell renal cell carcinoma	480	452	15.0	35.6	41.6	7.7
	Papillary renal cell carcinoma	163	138	23.9	38.4	18.1	19.6
	Clear cell papillary renal cell tumour	5	5	0.0	40.0	60.0	0.0
	Chromophobe renal cell carcinoma	89	66	3.0	6.1	12.1	78.8
	Oncocytoma	130	106	1.9	12.3	29.2	56.6
Tumors of the male genital organs	Adenocarcinoma of the prostate, Gleason 3 + 3	83	81	100.0	0.0	0.0	0.0
	Adenocarcinoma of the prostate, Gleason 4 + 4	80	73	100.0	0.0	0.0	0.0
	Adenocarcinoma of the prostate, Gleason 5 + 5	85	81	100.0	0.0	0.0	0.0
	Small cell neuroendocrine carcinoma of the prostate	17	16	100.0	0.0	0.0	0.0
	Seminoma	621	522	100.0	0.0	0.0	0.0
	Embryonal carcinoma of the testis	50	31	100.0	0.0	0.0	0.0
	Leydig cell tumor of the testis	30	28	100.0	0.0	0.0	0.0
	Sertoli cell tumor of the testis	2	2	100.0	0.0	0.0	0.0
	Sex cord stromal tumor of the testis	1	1	100.0	0.0	0.0	0.0
	Spermatocytic tumor of the testis	1	1	100.0	0.0	0.0	0.0
	Yolk sac tumor	50	38	100.0	0.0	0.0	0.0
	Teratoma	50	13	100.0	0.0	0.0	0.0
	Squamous cell carcinoma of the penis	80	70	100.0	0.0	0.0	0.0
Tumors of endocrine organs	Adenoma of the thyroid gland	113	94	13.8	10.6	5.3	70.2
	Papillary thyroid carcinoma	391	212	93.4	4.7	0.9	0.9
	Follicular thyroid carcinoma	154	67	40.3	25.4	7.5	26.9
	Medullary thyroid carcinoma	111	95	100.0	0.0	0.0	0.0
	Parathyroid gland adenoma	43	34	100.0	0.0	0.0	0.0
	Anaplastic thyroid carcinoma	45	38	100.0	0.0	0.0	0.0
	Adrenal cortical adenoma	50	30	93.3	3.3	3.3	0.0
	Adrenal cortical carcinoma	26	25	100.0	0.0	0.0	0.0
	Phaeochromocytoma	50	50	100.0	0.0	0.0	0.0
	Appendix, neuroendocrine tumor (NET)	22	19	100.0	0.0	0.0	0.0
	Colorectal, neuroendocrine tumor (NET)	12	11	90.9	9.1	0.0	0.0
	Ileum, neuroendocrine tumor (NET)	49	49	95.9	4.1	0.0	0.0
	Lung, neuroendocrine tumor (NET)	19	19	73.7	10.5	10.5	5.3
	Pancreas, neuroendocrine tumor (NET)	97	94	88.3	10.6	1.1	0.0
	Colorectal, neuroendocrine carcinoma (NEC)	12	11	100.0	0.0	0.0	0.0
	Gallbladder, neuroendocrine carcinoma (NEC)	4	4	75.0	0.0	25.0	0.0
	Pancreas, neuroendocrine carcinoma (NEC)	14	13	100.0	0.0	0.0	0.0
Tumors of haemotopoetic and lymphoid tissues	Hodgkin Lymphoma	103	101	100.0	0.0	0.0	0.0
	Small lymphocytic lymphoma, B-cell type (B-SLL/B-CLL)	50	48	100.0	0.0	0.0	0.0
	Diffuse large B cell lymphoma (DLBCL)	113	107	100.0	0.0	0.0	0.0
	Follicular lymphoma	88	80	100.0	0.0	0.0	0.0
	T-cell Non Hodgkin lymphoma	25	25	100.0	0.0	0.0	0.0
	Mantle cell lymphoma	18	17	100.0	0.0	0.0	0.0
	Marginal zone lymphoma	16	15	100.0	0.0	0.0	0.0
	Diffuse large B-cell lymphoma (DLBCL) in the testis	16	16	100.0	0.0	0.0	0.0
	Burkitt lymphoma	5	4	100.0	0.0	0.0	0.0
Tumors of soft tissue and bone	Tenosynovial giant cell tumor	45	39	100.0	0.0	0.0	0.0
	Granular cell tumor	53	47	100.0	0.0	0.0	0.0
	Leiomyoma	50	50	100.0	0.0	0.0	0.0
	Leiomyosarcoma	87	87	100.0	0.0	0.0	0.0
	Liposarcoma	132	126	100.0	0.0	0.0	0.0
	Malignant peripheral nerve sheath tumor (MPNST)	13	13	100.0	0.0	0.0	0.0
	Myofibrosarcoma	26	26	100.0	0.0	0.0	0.0
	Angiosarcoma	73	66	100.0	0.0	0.0	0.0
	Angiomyolipoma	91	90	100.0	0.0	0.0	0.0
	Dermatofibrosarcoma protuberans	21	18	100.0	0.0	0.0	0.0
	Ganglioneuroma	14	13	100.0	0.0	0.0	0.0
	Kaposi sarcoma	8	6	100.0	0.0	0.0	0.0
	Neurofibroma	117	86	100.0	0.0	0.0	0.0
	Sarcoma, not otherwise specified (NOS)	74	72	100.0	0.0	0.0	0.0
	Paraganglioma	41	38	100.0	0.0	0.0	0.0
	Ewing sarcoma	23	20	100.0	0.0	0.0	0.0
	Rhabdomyosarcoma	6	6	100.0	0.0	0.0	0.0
	Schwannoma	121	106	100.0	0.0	0.0	0.0
	Synovial sarcoma	12	11	100.0	0.0	0.0	0.0
	Osteosarcoma	43	37	100.0	0.0	0.0	0.0
	Chondrosarcoma	38	26	100.0	0.0	0.0	0.0
	Rhabdoid tumor	5	5	100.0	0.0	0.0	0.0

### CDH16 vs. Thyreoglobulin (TG) expression

The relationship between CDH16 expression and TG expression is shown in Supplementary Table 1. As TG expression was strictly limited to normal and neoplastic thyroidal epithelial cells^21^, dual positivity was commonly seen in benign thyroidal tissues, while the combination “CDH16 negative/TG positive” was strongly linked to thyroidal neoplasms and positivity for CDH16 alone was only seen in non-thyroidal neoplasms.

## Discussion

Our successful analysis of 13,424 tumors from 150 entities identified CDH16 expression in 40 of 152 analyzed tumor categories and enabled a ranking of tumor types according to their CDH16 positivity rate. The most commonly CDH16 positive cancers included renal cell carcinomas, nephrogenic adenomas, and follicular neoplasms of the thyroid, followed by tumors of the female genital tract and various categories of neuroendocrine tumors. This is largely consistent with RNA expression data from The Cancer Genome Atlas Research Network (https://www.cancer.gov/tcga), suggesting overwhelmingly high rates and levels of CDH16 expression in renal cell carcinomas and—less frequently and at lower levels—in carcinomas of the thyroid, endometrium, ovary, and the uterine cervix. These data suggest three diagnostic applications of CDH16 immunohistochemistry including a) diagnosing papillary thyroid cancer, b) the distinction of renal cell carcinomas from other tumors in case of metastases with unknown primary tumor or in renal masses where a urothelial carcinoma or a metastatic tumor remain diagnostic options, and c) the diagnosis of nephrogenic adenomas.

The histologic diagnosis of papillary carcinoma of the thyroid is less complex than of follicular carcinoma but many papillary neoplasms of the thyroid remain diagnostically challenging^22–25^. This especially applies to the more than 41% of papillary carcinomas that show a pure follicular growth pattern^26^. Lloyd et al. reported a concordance rate of only 39% between 10 expert pathologists for the follicular variant of papillary carcinomas^27^. Difficulties are even higher in cytology where the sensitivity is reported to be 40%-96% for recognizing papillary carcinomas^28–41^. Considering the unequivocal and strong CDH16 staining in all normal thyroid samples, as well as the CDH16 expression loss in more than 90% of our papillary carcinomas, CDH16 loss appears to constitute a strong argument in favor of a papillary carcinoma. The high rate of papillary thyroid cancers lacking CDH16 staining is consistent with data from Li et al.^4^, describing markedly lower CDH16 RNA levels in papillary carcinomas as compared to normal tissues in 505 patients from the TCGA dataset and in 16 own cases. The functional role of CDH16 expression loss in thyroid neoplasms is unclear. Koumarianou et al.^3^ found a role of CDH16 for the formation of follicular structures which are, however, at least partially retained in many papillary carcinomas. It is of note that Cali et al.^9^ also described a reduced CDH16 expression in follicular carcinomas. A possible role of reduced CDH16 expression for a subset of follicular thyroidal neoplasms is consistent with the 13.8% adenomas and the 40.3% follicular carcinomas with CDH16 negativity in this study. In a previous study on a subset of these tumors we had identified thyroglobulin (TG) as a highly specific and sensitive marker for follicular and papillary thyroid cancer which is, however, unable to distinguish benign from malignant throidal tissue^21^. A particular diagnostic value of CDH16 may exist for thyroidal tissue detected in cervical lymph nodes where benign thyroid inclusions (expected to be TG and CDH16 positive) and metastases (TG positive, often CDH16 negative) must be considered.

The high rate of CDH16 positive kidney tumors in our cohort fits with data from existing RNA databases (https://www.cancer.gov/tcga). In analogy to the higher staining intensity in distal than in proximal tubuli of the normal kidney, the CDH16 staining was generally more intense in oncocytomas and chromophobe carcinomas—both derived from distal tubuli—than in papillary and clear cell carcinomas arising from proximal tubuli^10,12,13^. All 8 previous studies analyzing CDH16 by immunohistochemistry in tumors were all limited to renal cell carcinomas^10–17^. They described CDH16 positivity in 0% to 95% of 6–41 analyzed oncocytomas ^10–14,17^, 5.6% to 100% of 7–36 chromophobe RCCs^10–15,17^, 0% to 29% of 14–46 papillary RCCs^10–13,16^, and 0% to 30% of 15–102 clear cell RCCs^10–15^. The rather high rate of CDH16 positive clear cell (85%) and papillary (76%) RCCs in our study as compared to earlier data appears to be due to a higher sensitivity of our IHC approach which may not have negatively affected its specificity based on the virtual absence of non-specific staining in normal tissues. Although CDH16 expression differences between renal cancer subtypes are statistically significant, our data do not suggest a relevant practical utility of CDH16 immunohistochemistry for subtype distinction at the selected experimental conditions. This is also because of the striking utility of CD117 for this distinction^42,43^. We previously found a significant link between low CDH16 expression and unfavorable tumor phenotype and poor prognosis in clear cell RCC which may argue for a functional role of CDH16 expression loss in the progression of these tumors^44^. The high sensitivity of our assay may also be responsible for the detection of a significant CDH16 staining in 12 of 12 nephrogenic adenomas while Ortiz-Rey et al.^45^ had described CDH16 positivity in only 9 of 12 cases. Whether some of the novel oncocytic and molecularly defined RCC subtypes (eosinophilic vacuolated tumour, low-grade oncocytic tumour and TFE3-rearranged, TFEB-altered, ELOC (formerly TCEB1)-mutated, fumarate hydratase-deficient, succinate dehydrogenase-deficient, ALK-rearranged renal cell carcinomas and SMARCB1-deficient renal medullary carcinoma)—which were not distinguished in our historic tumor collection—may be particularly linked to CDH16 negativity needs to be determined in further studies.

Our data suggest that CDH16 immunohistochemistry may be useful for the distinction of renal cell carcinomas from other neoplasms. Although CDH16 is not specific for renal cell carcinomas and can also be seen in gynecological, neuroendocrine and several other tumors, it is noteworthy that CDH16 expression is mostly weak and not involving all cells in these extrarenal neoplasms. Completely renal specific antibodies have so far not been discovered. Immunohistochemical markers that are most commonly used for the distinction of renal cell carcinomas include PAX8 and CAIX^46–48^. However, PAX8 is abundantly expressed in gynecological tumors^49–51^ and thyroid cancers^52–54^, and can be found in various other tumors as well^55–58^. CAIX lacks expression in chromophobe kidney cancer^59^ and can also be expressed at high levels in various extrarenal tumors^60–65^. Studies are now needed to determine to what extent the additional use of CDH16 will improve the diagnostic precision of panels applied for establishing a renal cell tumor origin. The same applies for a potential diagnostic utility of CDH16 IHC in endocervical adenocarcinomas. Given the conspicuously high rate of CDH16 positive cervical adenocarcinomas as compared to the paucity of CDH16 positive cells in normal endocervical epithelium, significant CDH16 positivity may argue for malignancy at this location. The strong CDH16 positivity in mesonephric duct remnants must be considered, however, as these do regularly also occur in the uterine cervix^66^.

Considering the large scale of our study, our assay was extensively validated by comparing our IHC findings in normal tissues with data obtained by another independent anti-CDH16 antibody and RNA data derived from three different publicly accessible databases^5–8^. To ensure that the widest possible range of proteins would be tested for a possible cross-reactivity, 76 different normal tissues categories were included in this analysis. Validity of our assay was supported by the detection of significant CDH16 immunostaining in all organs with documented CDH16 RNA expression (thyroid, kidney, epididymis, seminal vesicles, and the fallopian tube). Additional CDH16 staining in gallbladder epithelium, the uterine cervix, endometrium glands, or mesonephric remnants, for which CDH16 RNA expression had not been described, were confirmed by the independent second antibody (Abcam EPR13090). In these organs, the CDH16 positive cells constitute such small fraction of the total number of cells that CDH16 RNA may not be present at detectable quantities in usual tissue samples.

Our data provide a comprehensive overview on CDH16 expression in normal and neoplastic human tissues. These findings suggest that—as a part of a panel—CDH16 immunohistochemistry might assist the identification of papillary thyroid cancer, the distinction of renal cell carcinomas from other neoplasms in cases of uncertain tumor origin, and the diagnosis of a nephrogenic adenoma.

## Material and methods

### Tissue microarrays (TMAs)

The normal tissue TMA was composed of 8 samples from 8 different donors for each of 76 different normal tissue types (608 samples on one slide). The cancer TMAs contained a total of 15,873 primary tumors from 150 tumor types and subtypes. The composition of both normal and tumor TMAs is described in detail in the results section. All samples were from the archives of the Institutes of Pathology, University Hospital of Hamburg, Germany, the Institute of Pathology, Clinical Center Osnabrueck, Germany, and Department of Pathology, Academic Hospital Fuerth, Germany. Tissues were fixed in 4% buffered formalin and then embedded in paraffin. The TMA manufacturing process was described earlier in detail^67,68^. In brief, one tissue spot (diameter: 0.6 mm) was transmitted from a tumor containing donor block in an empty recipient paraffin block. The use of archived remnants of diagnostic tissues for manufacturing of TMAs and their analysis for research purposes as well as patient data analysis has been approved by local laws (HmbKHG, §12) and by the local ethics committee (Ethics commission Hamburg, WF-049/09). All work has been carried out in compliance with the Helsinki Declaration. For data confirmation and extension, large section analyses were also executed on 10 cases each of normal thyroid, endocervix, fallopian tube, and gallbladder, 12 nephrogenic adenomas of the urinary bladder, as well as on 3 peritoneal and 2 mesotheliomas of the tunica albuginea. Data on thyroglobulin (TG) immunostaining were available for a subset of 8643 of our tumors from a previous study^21^.

### Immunohistochemistry (IHC)

Freshly prepared TMA sections were immunostained on one day in one experiment. Slides were deparaffinized with xylol, rehydrated through a graded alcohol series and exposed to heat-induced antigen retrieval for 5 min in an autoclave at 121 °C in pH 7.8 DakoTarget Retrieval Solution™ (Agilent, CA, USA; #S2367). Endogenous peroxidase activity was blocked with Dako Peroxidase Blocking Solution™ (Agilent, CA, USA; #52023) for 10 min. Primary antibody specific against CDH16 protein (Recombinant monoclonal rabbit, MSVA-516R, MS Validated Antibodies, Hamburg, Germany) was applied at 37 °C for 60 min at a dilution of 1:150. For the purpose of antibody validation, the normal tissue TMA was also analyzed by the monoclonal rabbit CDH16 antibody [EPR13090] (Abcam; Cambridge, United Kingdom, ab214092) at a dilution of 1:300 and an otherwise identical protocol. Bound antibody was visualized using the EnVision Kit™ (Agilent, CA, USA; #K5007) according to the manufacturer’s directions. The sections were counterstained with haemalaun. For normal tissues, the staining intensity of positive cells was semi-quantitively recorded (+ , +  + , +  + +). For tumor tissues, the percentage of CDH16 positive tumor cells was estimated and the staining intensity was semi-quantitatively recorded (0, 1 + , 2 + , 3 +). For statistical analyses, the staining results were categorized into four groups as follows: Negative: no staining at all, weak staining: staining intensity of 1 + in ≤ 70% or staining intensity of 2 + in ≤ 30% of tumor cells, moderate staining: staining intensity of 1 + in > 70%, staining intensity of 2 + in > 30% but in ≤ 70% or staining intensity of 3 + in ≤ 30% of tumor cells, strong staining: staining intensity of 2 + in > 70% or staining intensity of 3 + in > 30% of tumor cells.

### Statistics

Statistical calculations were performed with JMP 14 software (SAS Institute Inc., NC, USA). Contingency tables and the chi^2^-test were performed to search for associations between CDH16 expression and tumor phenotype. A p-value ≤ 0.05 was considered significant.

### Ethics declarations

The study was approved by the Ethics commission Hamburg (WF-049/09) and conducted in accordance with the Declaration of Helsinki. Informed consent has not been collected specifically for the patient samples included in this study. Usage of routinely archived formalin fixed leftover patient tissue samples for research purposes by the attending physician is approved by local laws and does not require written consent (HmbKHG, §12,1).

### Supplementary Information

Supplementary Figure 1.

Supplementary Table 1.

## Data Availability

All data generated or analyzed during this study are included in this published article.

## References

[1] Thedieck C (2005). Expression of Ksp-cadherin during kidney development and in renal cell carcinoma. Br. J. Cancer.

[2] Thomson RB, Aronson PS (1999). Immunolocalization of Ksp-cadherin in the adult and developing rabbit kidney. Am. J. Physiol..

[3] Koumarianou P, Gomez-Lopez G, Santisteban P (2017). Pax8 controls thyroid follicular polarity through cadherin-16. J. Cell Sci..

[4] Li P (2019). Downregulation of CDH16 in papillary thyroid cancer and its potential molecular mechanism analysed by qRT-PCR, TCGA and in silico analysis. Cancer Manag. Res..

[5] Lizio M (2019). Update of the FANTOM web resource: Expansion to provide additional transcriptome atlases. Nucleic Acids Res..

[6] Thul PJ (2017). A subcellular map of the human proteome. Science.

[7] Lizio M (2015). Gateways to the FANTOM5 promoter level mammalian expression atlas. Genome Biol..

[8] Consortium GT (2013). The Genotype-Tissue Expression (GTEx) project. Nat. Genet..

[9] Cali G (2012). CDH16/Ksp-cadherin is expressed in the developing thyroid gland and is strongly down-regulated in thyroid carcinomas. Endocrinology.

[10] Kuehn A (2007). Expression analysis of kidney-specific cadherin in a wide spectrum of traditional and newly recognized renal epithelial neoplasms: Diagnostic and histogenetic implications. Am. J. Surg. Pathol..

[11] Adley BP (2006). Expression of kidney-specific cadherin in chromophobe renal cell carcinoma and renal oncocytoma. Am. J. Clin. Pathol..

[12] Mazal PR (2005). Expression of kidney-specific cadherin distinguishes chromophobe renal cell carcinoma from renal oncocytoma. Hum. Pathol..

[13] Shen SS, Krishna B, Chirala R, Amato RJ, Truong LD (2005). Kidney-specific cadherin, a specific marker for the distal portion of the nephron and related renal neoplasms. Mod. Pathol..

[14] Yasir S (2012). CD10+ and CK7/RON- immunophenotype distinguishes renal cell carcinoma, conventional type with eosinophilic morphology from its mimickers. Appl. Immunohistochem. Mol. Morphol..

[15] Iribe Y (2015). Immunohistochemical characterization of renal tumors in patients with Birt-Hogg-Dube syndrome. Pathol. Int..

[16] Han G (2017). Oncocytic papillary renal cell carcinoma: A clinicopathological and genetic analysis and indolent clinical course in 14 cases. Pathol. Res. Pract..

[17] Gaut JP, Crimmins DL, Lockwood CM, McQuillan JJ, Ladenson JH (2013). Expression of the Na+/K+-transporting ATPase gamma subunit FXYD2 in renal tumors. Mod. Pathol..

[18] Bloomstein JD (2020). Validated limited gene predictor for cervical cancer lymph node metastases. Oncotarget.

[19] Li WB (2016). Identification of genes associated with papillary thyroid carcinoma (PTC) for diagnosis by integrated analysis. Horm. Metab. Res..

[20] Fontaine JF (2009). Increasing the number of thyroid lesions classes in microarray analysis improves the relevance of diagnostic markers. PLoS One.

[21] Steurer S (2021). Immunohistochemically detectable thyroglobulin expression in extrathyroidal cancer is 100% specific for thyroidal tumor origin. Ann. Diagn. Pathol..

[22] Wallander M (2010). Follicular variant of papillary carcinoma: Reproducibility of histologic diagnosis and utility of HBME-1 immunohistochemistry and BRAF mutational analysis as diagnostic adjuncts. Appl. Immunohistochem. Mol. Morphol..

[23] Gupta S, Sodhani P, Jain S, Kumar N (2004). Morphologic spectrum of papillary carcinoma of the thyroid: Role of cytology in identifying the variants. Acta Cytol..

[24] Franc B (2003). Interobserver and intraobserver reproducibility in the histopathology of follicular thyroid carcinoma. Hum. Pathol..

[25] Saxen E, Franssila K, Bjarnason O, Normann T, Ringertz N (1978). Observer variation in histologic classification of thyroid cancer. Acta Pathol. Microbiol. Scand. A.

[26] Zidan J (2003). Pure versus follicular variant of papillary thyroid carcinoma: Clinical features, prognostic factors, treatment, and survival. Cancer.

[27] Lloyd RV (2004). Observer variation in the diagnosis of follicular variant of papillary thyroid carcinoma. Am. J. Surg. Pathol..

[28] Sulejmanovic M, Cickusic AJ, Salkic S (2019). The value of fine-needle aspiration biopsy (FNAB) in differential diagnosis of scintigraphic cold thyroid nodule. Acta Inform. Med..

[29] Seningen JL, Nassar A, Henry MR (2012). Correlation of thyroid nodule fine-needle aspiration cytology with corresponding histology at Mayo Clinic, 2001–2007: An institutional experience of 1,945 cases. Diagn. Cytopathol..

[30] Proietti A (2014). Molecular characterization of 54 cases of false-negative fine-needle aspiration among 1347 papillary thyroid carcinomas. Cancer Cytopathol..

[31] Jo VY, Renshaw AA, Krane JF (2013). Relative sensitivity of thyroid fine-needle aspiration by tumor type and size. Diagn. Cytopathol..

[32] Mehanna R (2013). False negatives in thyroid cytology: Impact of large nodule size and follicular variant of papillary carcinoma. Laryngoscope.

[33] Redlich A (2012). Sensitivity of fine-needle biopsy in detecting pediatric differentiated thyroid carcinoma. Pediatr. Blood Cancer.

[34] Adeniran AJ (2011). Reflex BRAF testing in thyroid fine-needle aspiration biopsy with equivocal and positive interpretation: A prospective study. Thyroid.

[35] Bargren AE (2010). Diagnostic utility of fine-needle aspiration cytology in pediatric differentiated thyroid cancer. World J. Surg..

[36] de la Serna Saravia C, Cuellar F, Saravio Day E, Harach HR (2006). Accuracy of aspiration cytology in thyroid cancer: A study in 1 institution. Acta Cytol..

[37] Furlan JC, Bedard YC, Rosen IB (2004). Role of fine-needle aspiration biopsy and frozen section in the management of papillary thyroid carcinoma subtypes. World J. Surg..

[38] Yeh MW, Demircan O, Ituarte P, Clark OH (2004). False-negative fine-needle aspiration cytology results delay treatment and adversely affect outcome in patients with thyroid carcinoma. Thyroid.

[39] Munn JS, Castelli M, Prinz RA, Walloch JL (1988). Needle biopsy of nodular thyroid disease. Am. Surg..

[40] Akerman M, Tennvall J, Biorklund A, Martensson H, Moller T (1985). Sensitivity and specificity of fine needle aspiration cytology in the diagnosis of tumors of the thyroid gland. Acta Cytol..

[41] Radetic M, Kralj Z, Padovan I (1984). Reliability of aspiration biopsy in thyroid nodes: Study of 2190 operated patients. Tumori.

[42] Chen CV, Croom NA, Simko JP, Stohr BA, Chan E (2022). Differential immunohistochemical and molecular profiling of conventional and aggressive components of chromophobe renal cell carcinoma: Pitfalls for diagnosis. Hum. Pathol..

[43] Shen SS, Truong LD, Scarpelli M, Lopez-Beltran A (2012). Role of immunohistochemistry in diagnosing renal neoplasms: When is it really useful?. Arch. Pathol. Lab. Med..

[44] Lennartz M (2022). Reduced CDH16 expression is linked to poor prognosis in clear cell renal cell carcinoma 16. Urol. Oncol..

[45] Ortiz-Rey JA, Anton-Badiola I, Perez-Pedrosa A, Peteiro-Cancelo A, Gonzalez-Carrero J (2012). Nephrogenic adenoma: An immunohistochemical analysis using biotin-free methods. Appl. Immunohistochem. Mol. Morphol..

[46] Courcier J (2020). Carbonic anhydrase IX in renal cell carcinoma, implications for disease management. Int. J. Mol. Sci..

[47] Alshenawy HA (2015). Immunohistochemical panel for differentiating renal cell carcinoma with clear and papillary features. Pathol. Oncol. Res..

[48] Yu W (2013). Clinicopathological, genetic, ultrastructural characterizations and prognostic factors of papillary renal cell carcinoma: New diagnostic and prognostic information. Acta Histochem..

[49] Chai HJ (2017). PAX8 is a potential marker for the diagnosis of primary epithelial ovarian cancer. Oncol. Lett..

[50] Rodgers LH, Young AN, Burdette JE (2016). Loss of PAX8 in high-grade serous ovarian cancer reduces cell survival despite unique modes of action in the fallopian tube and ovarian surface epithelium. Oncotarget.

[51] Zhao L, Guo M, Sneige N, Gong Y (2012). Value of PAX8 and WT1 immunostaining in confirming the ovarian origin of metastatic carcinoma in serous effusion specimens. Am. J. Clin. Pathol..

[52] Rosignolo F (2016). Expression of PAX8 target genes in papillary thyroid carcinoma. PLoS One.

[53] Suzuki A (2015). Diagnostic significance of PAX8 in thyroid squamous cell carcinoma. Endocr. J..

[54] Nonaka D, Tang Y, Chiriboga L, Rivera M, Ghossein R (2008). Diagnostic utility of thyroid transcription factors Pax8 and TTF-2 (FoxE1) in thyroid epithelial neoplasms. Mod. Pathol..

[55] Lu H, Allende D, Liu X, Zhang Y (2020). Lymphoid enhancer binding factor 1 (LEF1) and paired box gene 8 (PAX8): A limited immunohistochemistry panel to distinguish solid pseudopapillary neoplasms and pancreatic neuroendocrine tumors. Appl. Immunohistochem. Mol. Morphol..

[56] Chen YB, Fine SW, Epstein JI (2011). Mesonephric remnant hyperplasia involving prostate and periprostatic tissue: Findings at radical prostatectomy. Am. J. Surg. Pathol..

[57] Corben AD, Nehhozina T, Garg K, Vallejo CE, Brogi E (2010). Endosalpingiosis in axillary lymph nodes: A possible pitfall in the staging of patients with breast carcinoma. Am. J. Surg. Pathol..

[58] Tong GX (2008). Expression of PAX8 in nephrogenic adenoma and clear cell adenocarcinoma of the lower urinary tract: Evidence of related histogenesis?. Am. J. Surg. Pathol..

[59] Buscheck F (2018). Aberrant expression of membranous carbonic anhydrase IX (CAIX) is associated with unfavorable disease course in papillary and clear cell renal cell carcinoma. Urol. Oncol..

[60] Alves W (2019). CAIX is a predictor of pathological complete response and is associated with higher survival in locally advanced breast cancer submitted to neoadjuvant chemotherapy. BMC Cancer.

[61] Eckert AW (2019). Investigation of the prognostic role of carbonic anhydrase 9 (CAIX) of the cellular mRNA/protein level or soluble CAIX protein in patients with oral squamous cell carcinoma. Int. J. Mol. Sci..

[62] Yang L (2018). Overexpression of FZD1 and CAIX are associated with invasion, metastasis, and poor-prognosis of the pancreatic ductal adenocarcinoma. Pathol. Oncol. Res..

[63] Senol S (2016). Gastric adenocarcinoma biomarker expression profiles and their prognostic value. J. Environ. Pathol. Toxicol. Oncol..

[64] Pinheiro C (2011). GLUT1 and CAIX expression profiles in breast cancer correlate with adverse prognostic factors and MCT1 overexpression. Histol. Histopathol..

[65] Hyuga S (2017). Expression of carbonic anhydrase IX is associated with poor prognosis through regulation of the epithelialmesenchymal transition in hepatocellular carcinoma. Int. J. Oncol..

[66] Meguro S, Yasuda M, Shimizu M, Kurosaki A, Fujiwara K (2013). Mesonephric adenocarcinoma with a sarcomatous component, a notable subtype of cervical carcinosarcoma: A case report and review of the literature. Diagn. Pathol..

[67] Kononen J (1998). Tissue microarrays for high-throughput molecular profiling of tumor specimens. Nat. Med..

[68] Dancau AM, Simon R, Mirlacher M, Sauter G (2016). Tissue microarrays. Methods Mol. Biol..

