# Supplementary material for: Cadherin-16 (CDH16) immunohistochemistry: a useful diagnostic tool for renal cell carcinoma and papillary carcinomas of the thyroid
Source: Sci Rep. 2023 Aug 9;13:12917. doi: 10.1038/s41598-023-39945-2 (PMC10412623; doi:10.1038/s41598-023-39945-2)
Supplement: Supplementary file 1 — Supplementary Figure 1. [file 41598_2023_39945_MOESM1_ESM.pdf]

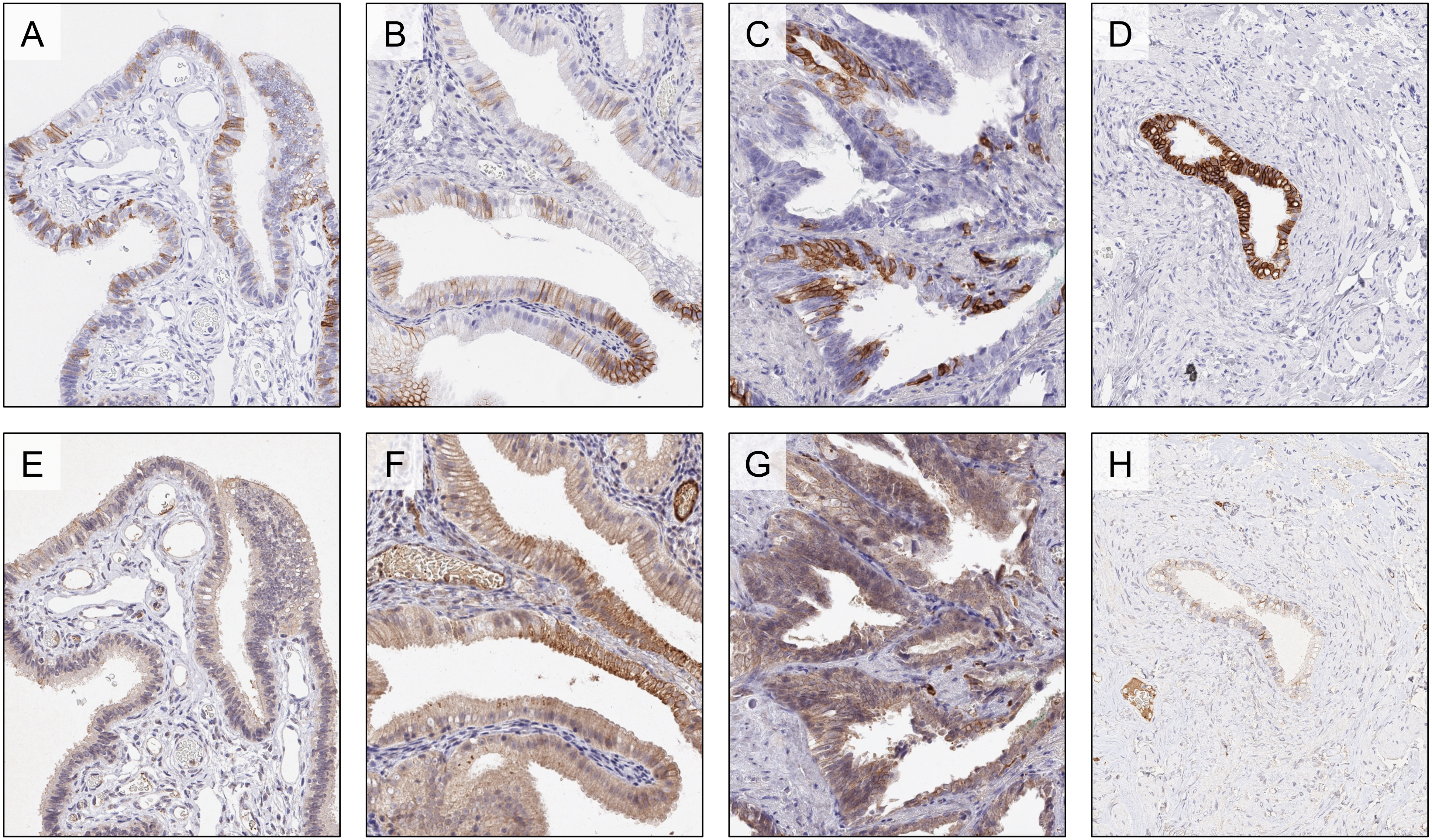

Suppl Fig. 1. IHC validation by comparison of antibodies. The panels show a concordance of immunostaining results obtained by two independent CDH16 antibodies. Using MSVA-516R, the stainings show a predominantly membranous positivity of scattered epithelial cells of the fallopian tube (A), epithelial cells of the gallbladder (B), groups of epithelial cells in the seminal vesicle (C), and in a mesonephric remnant of the fallopian tube (D). Using clone [EPR13090] , a comparable staining is seen in the fallopian tube (E), gallbladder (F), seminal vesicle (G), and the mesonephric remnant (H). The images A-D and E-H are from consecutive tissue sections.
