# Supplementary material for: Cadherin-16 (CDH16) immunohistochemistry: a useful diagnostic tool for renal cell carcinoma and papillary carcinomas of the thyroid
Source: Sci Rep. 2023 Aug 9;13:12917. doi: 10.1038/s41598-023-39945-2 (PMC10412623; doi:10.1038/s41598-023-39945-2)
Supplement: Supplementary file 2 — Supplementary Table 1. [file 41598_2023_39945_MOESM2_ESM.pdf]

Supplemental table 1: CDH16 and Thyroglobulin immunostaining in human tumors.

	Tumor entity	on TMA (n)	CDH16 and Thyroglobulin immunostaining				
			analyzable (n)	CDH16 and Thyroglobulin negative (%)	CDH16 positive / Thyroglobulin negative (%)	Thyroglobulin positive / CDH16 negative (%)	CDH16 and Thyroglobulin positive (%)
**Tumors of the skin**	Pilomatrixoma	35	31	100,0	0,0	0,0	0,0
	Basal cell carcinoma	88	66	100,0	0,0	0,0	0,0
	Benign nevus	29	26	100,0	0,0	0,0	0,0
	Squamous cell carcinoma of the skin	90	86	100,0	0,0	0,0	0,0
	Malignant melanoma	46	40	100,0	0,0	0,0	0,0
	Merkel cell carcinoma	46	41	100,0	0,0	0,0	0,0
**Tumors of the head and neck**	Squamous cell carcinoma of the larynx	109	99	99,0	1,0	0,0	0,0
	Squamous cell carcinoma of the pharynx	60	52	100,0	0,0	0,0	0,0
	Oral squamous cell carcinoma (floor of the mouth)	130	118	100,0	0,0	0,0	0,0
	Pleomorphic adenoma of the parotid gland	50	44	100,0	0,0	0,0	0,0
	Warthin tumor of the parotid gland	49	48	100,0	0,0	0,0	0,0
	Basal cell adenoma of the salivary gland	15	15	100,0	0,0	0,0	0,0
**Tumors of the lung, pleura and thymus**	Adenocarcinoma of the lung	246	98	100,0	0,0	0,0	0,0
	Squamous cell carcinoma of the lung	130	38	100,0	0,0	0,0	0,0
	Small cell carcinoma of the lung	20	14	100,0	0,0	0,0	0,0
	Mesothelioma, epitheloid	39	31	100,0	0,0	0,0	0,0
	Mesothelioma, other types	76	58	100,0	0,0	0,0	0,0
	Thymoma	29	29	100,0	0,0	0,0	0,0
**Tumors of the female genital tract**	Squamous cell carcinoma of the vagina	78	56	100,0	0,0	0,0	0,0
	Squamous cell carcinoma of the vulva	130	108	100,0	0,0	0,0	0,0
	Squamous cell carcinoma of the cervix	129	111	100,0	0,0	0,0	0,0
	Endometrioid endometrial carcinoma	236	172	81,4	18,6	0,0	0,0
	Endometrial serous carcinoma	82	55	61,8	38,2	0,0	0,0
	Carcinosarcoma of the uterus	48	27	92,6	7,4	0,0	0,0
	Endometrial carcinoma, high grade, G3	13	7	100,0	0,0	0,0	0,0
	Endometrial clear cell carcinoma	8	3	66,7	33,3	0,0	0,0
	Endometrioid carcinoma of the ovary	110	71	87,3	12,7	0,0	0,0
	Serous carcinoma of the ovary	559	414	94,2	5,8	0,0	0,0
	Mucinous carcinoma of the ovary	96	57	61,4	38,6	0,0	0,0
	Clear cell carcinoma of the ovary	50	36	41,7	58,3	0,0	0,0

	Tumor entity	on TMA (n)	CDH16 and Thyroglobulin immunostaining				
			analyzable (n)	CDH16 and Thyroglobulin negative (%)	CDH16 positive / Thyroglobulin negative (%)	Thyroglobulin positive / CDH16 negative (%)	CDH16 and Thyroglobulin positive (%)
	Carcinosarcoma of the ovary	47	32	81,3	18,8	0,0	0,0
	Brenner tumor	9	7	100,0	0,0	0,0	0,0
**Tumors of the breast**	Invasive breast carcinoma of no special type	126	51	100,0	0,0	0,0	0,0
	Lobular carcinoma of the breast	123	77	100,0	0,0	0,0	0,0
	Medullary carcinoma of the breast	15	13	100,0	0,0	0,0	0,0
	Tubular carcinoma of the breast	18	13	100,0	0,0	0,0	0,0
	Mucinous carcinoma of the breast	22	15	100,0	0,0	0,0	0,0
	Phyllodes tumor of the breast	50	42	100,0	0,0	0,0	0,0
**Tumors of the digestive system**	Adenomatous polyp, low-grade dysplasia	50	47	100,0	0,0	0,0	0,0
	Adenomatous polyp, high-grade dysplasia	50	46	100,0	0,0	0,0	0,0
	Adenocarcinoma of the colon	1932	1296	98,8	1,2	0,0	0,0
	Gastric adenocarcinoma, diffuse type	226	128	96,9	3,1	0,0	0,0
	Gastric adenocarcinoma, intestinal type	224	130	96,9	3,1	0,0	0,0
	Gastric adenocarcinoma, mixed type	62	52	92,3	7,7	0,0	0,0
	Adenocarcinoma of the esophagus	133	59	100,0	0,0	0,0	0,0
	Squamous cell carcinoma of the esophagus	125	48	100,0	0,0	0,0	0,0
	Squamous cell carcinoma of the anal canal	89	80	100,0	0,0	0,0	0,0
	Cholangiocarcinoma	50	41	95,1	4,9	0,0	0,0
	Hepatocellular carcinoma	50	50	100,0	0,0	0,0	0,0
	Ductal adenocarcinoma of the pancreas	662	387	98,4	1,6	0,0	0,0
	Pancreatic/Ampullary adenocarcinoma	119	57	94,7	5,3	0,0	0,0
	Acinar cell carcinoma of the pancreas	16	13	92,3	7,7	0,0	0,0
	Gastrointestinal stromal tumor (GIST)	50	46	100,0	0,0	0,0	0,0
**Tumors of the urinary system**	Non-invasive papillary urothelial carcinoma, pTa G2 low grade	177	123	100,0	0,0	0,0	0,0
	Non-invasive papillary urothelial carcinoma, pTa G2 high grade	141	110	100,0	0,0	0,0	0,0
	Non-invasive papillary urothelial carcinoma, pTa G3	187	138	100,0	0,0	0,0	0,0
	Urothelial carcinoma, pT2-4 G3	623	416	99,5	0,5	0,0	0,0
	Small cell neuroendocrine carcinoma of the bladder	19	19	100,0	0,0	0,0	0,0
	Sarcomatoid urothelial carcinoma	25	24	100,0	0,0	0,0	0,0
	Clear cell renal cell carcinoma	50	32	21,9	78,1	0,0	0,0
	Papillary renal cell carcinoma	50	27	11,1	88,9	0,0	0,0
	Chromophobe renal cell carcinoma	50	27	0,0	100,0	0,0	0,0

	Tumor entity	on TMA (n)	CDH16 and Thyroglobulin immunostaining				
			analyzable (n)	CDH16 and Thyroglobulin negative (%)	CDH16 positive / Thyroglobulin negative (%)	Thyroglobulin positive / CDH16 negative (%)	CDH16 and Thyroglobulin positive (%)
	Oncocytoma	50	27	3,7	96,3	0,0	0,0
**Tumors of the male genital organs**	Adenocarcinoma of the prostate, Gleason 3+3	83	68	100,0	0,0	0,0	0,0
	Adenocarcinoma of the prostate, Gleason 4+4	80	63	100,0	0,0	0,0	0,0
	Adenocarcinoma of the prostate, Gleason 5+5	85	67	100,0	0,0	0,0	0,0
	Small cell neuroendocrine carcinoma of the prostate	17	16	100,0	0,0	0,0	0,0
	Seminoma	621	437	100,0	0,0	0,0	0,0
	Embryonal carcinoma of the testis	50	29	100,0	0,0	0,0	0,0
	Yolk sac tumor	50	30	100,0	0,0	0,0	0,0
	Teratoma	50	10	100,0	0,0	0,0	0,0
	Squamous cell carcinoma of the penis	80	68	100,0	0,0	0,0	0,0
**Tumors of endocrine organs**	Adenoma of the thyroid gland	113	92	1,1	9,8	13,0	76,1
	Papillary thyroid carcinoma	391	207	5,8	0,0	87,9	6,3
	Follicular thyroid carcinoma	154	66	3,0	6,1	36,4	54,5
	Medullary thyroid carcinoma	111	93	78,5	0,0	21,5	0,0
	Anaplastic thyroid carcinoma	45	38	94,7	0,0	5,3	0,0
	Adrenal cortical adenoma	50	29	93,1	6,9	0,0	0,0
	Adrenal cortical carcinoma	26	25	100,0	0,0	0,0	0,0
	Phaeochromocytoma	50	50	100,0	0,0	0,0	0,0
	Appendix, neuroendocrine tumor (NET)	22	14	100,0	0,0	0,0	0,0
	Colorectal, neuroendocrine tumor (NET)	12	11	90,9	9,1	0,0	0,0
	Ileum, neuroendocrine tumor (NET)	49	47	95,7	4,3	0,0	0,0
	Lung, neuroendocrine tumor (NET)	19	18	72,2	27,8	0,0	0,0
	Pancreas, neuroendocrine tumor (NET)	97	75	88,0	12,0	0,0	0,0
	Colorectal, neuroendocrine carcinoma (NEC)	12	11	100,0	0,0	0,0	0,0
	Gallbladder, neuroendocrine carcinoma (NEC)	4	4	75,0	25,0	0,0	0,0
	Pancreas, neuroendocrine carcinoma (NEC)	14	13	100,0	0,0	0,0	0,0
**Tumors of haemotopoetic and lymphoid tissues**	Hodgkin Lymphoma	103	78	100,0	0,0	0,0	0,0
	Small lymphocytic lymphoma, B-cell type (B-SLL/B-CLL)	50	48	100,0	0,0	0,0	0,0
	Diffuse large B cell lymphoma (DLBCL)	113	102	100,0	0,0	0,0	0,0
	Follicular lymphoma	88	79	100,0	0,0	0,0	0,0
	T-cell Non Hodgkin lymphoma	25	25	100,0	0,0	0,0	0,0
	Mantle cell lymphoma	18	17	100,0	0,0	0,0	0,0

	Tumor entity	on TMA (n)	CDH16 and Thyroglobulin immunostaining				
			analyzable (n)	CDH16 and Thyroglobulin negative (%)	CDH16 positive / Thyroglobulin negative (%)	Thyroglobulin positive / CDH16 negative (%)	CDH16 and Thyroglobulin positive (%)
	Marginal zone lymphoma	16	15	100,0	0,0	0,0	0,0
	Diffuse large B-cell lymphoma (DLBCL) in the testis	16	16	100,0	0,0	0,0	0,0
	Burkitt lymphoma	5	4	100,0	0,0	0,0	0,0
**Tumors of soft tissue and bone**	Tenosynovial giant cell tumor	45	39	100,0	0,0	0,0	0,0
	Granular cell tumor	53	46	100,0	0,0	0,0	0,0
	Leiomyoma	50	49	100,0	0,0	0,0	0,0
	Leiomyosarcoma	87	86	100,0	0,0	0,0	0,0
	Liposarcoma	132	122	100,0	0,0	0,0	0,0
	Malignant peripheral nerve sheath tumor (MPNST)	13	11	100,0	0,0	0,0	0,0
	Myofibrosarcoma	26	26	100,0	0,0	0,0	0,0
	Angiosarcoma	73	64	100,0	0,0	0,0	0,0
	Angiomyolipoma	91	88	100,0	0,0	0,0	0,0
	Dermatofibrosarcoma protuberans	21	16	100,0	0,0	0,0	0,0
	Ganglioneuroma	14	13	100,0	0,0	0,0	0,0
	Kaposi sarcoma	8	5	100,0	0,0	0,0	0,0
	Neurofibroma	117	86	100,0	0,0	0,0	0,0
	Sarcoma, not otherwise specified (NOS)	74	72	100,0	0,0	0,0	0,0
	Paraganglioma	41	38	100,0	0,0	0,0	0,0
	Ewing sarcoma	23	19	100,0	0,0	0,0	0,0
	Rhabdomyosarcoma	6	6	100,0	0,0	0,0	0,0
	Schwannoma	121	106	100,0	0,0	0,0	0,0
	Synovial sarcoma	12	11	100,0	0,0	0,0	0,0
	Osteosarcoma	43	34	100,0	0,0	0,0	0,0
	Chondrosarcoma	38	14	100,0	0,0	0,0	0,0
